# Hyperuricemia and the risk for subclinical coronary atherosclerosis - data from a prospective observational cohort study

**DOI:** 10.1186/ar3322

**Published:** 2011-04-18

**Authors:** Eswar Krishnan, Bhavik J Pandya, Lorinda Chung, Omar Dabbous

**Affiliations:** 1 Department of Medicine, Stanford University School of Medicine, 1000 Welch Road, Suite 203, Palo Alto, CA 94304, USA; 2 Department of Global Health Economics and Outcomes Research, Takeda Pharmaceuticals International, Inc., One Takeda Parkway, Deerfield, IL 60015, USA

## Abstract

**Introduction:**

Our purpose was to test the hypothesis that hyperuricemia is associated with coronary artery calcification (CAC) among a relatively healthy population, and that the extent of calcification is directly proportional to the serum uric acid (sUA) concentration.

**Methods:**

Data from 2,498 participants in the Coronary Artery Risk Development in Young Adults (CARDIA) study were analyzed using logistic regression models. Subjects were free of clinical heart disease, diabetes, and renal impairment. The main measure was the presence of any CAC by computerized tomography (Agatston score >0).

**Results:**

Forty-eight percent of the study participants were male and 45% were African-American. Mean (± SD) age was 40 ± 4 years, body mass index 28 ± 6 kg/m^2^, Framingham risk score -0.7 ± 5%, blood pressure 113 ± 14/75 ± 11 mmHg, alcohol consumption 12 ± 27 ml/day, and sUA 297 ± 89 μmol/L (5.0 ± 1.5 mg/dL). Prevalence of CAC increased with sUA concentration among both men and women. Adjusted for age, gender, race, lipoproteins, triglycerides, smoking, blood pressure, presence of metabolic syndrome, C-reactive protein, waist circumference, alcohol use, creatinine, and serum albumin, the highest quartile of sUA (>393 μmol/L [6.6 mg/dL] for men and >274 μmol/L [4.6 mg/dL] for women) was associated with an odds ratio of 1.87 (1.19-2.93) compared to the lowest quartile (<291 μmol/L [4.9 mg/dL] for men and <196 μmol/L [3.3 mg/dL] for women). Among those with any CAC, each unit increase in sUA was associated with a 22% increase in Agatston score (*P *= 0.008) after adjusting for the above covariates.

**Conclusions:**

Hyperuricemia is an independent risk factor for subclinical atherosclerosis in young adults.

## Introduction

Although the link between elevated serum uric acid (sUA) concentrations and the risk for atherosclerotic cardiovascular and cerebrovascular disease has long been observed, only recently have the pathophysiologic links become clearer [1]. Kanbay and colleagues [2] recently summarized the emerging data suggesting that hyperuricemia may cause not only atherosclerosis in the macrovascular beds such as the coronaries and the carotids but also microvascular damage in the renal vascular bed and may exacerbate vascular disease.

Almost all epidemiological studies performed in populations of higher-than-normal risk have shown a consistent association between sUA and coronary artery disease (CAD) [1]. Studies on the lower-than-normal-risk populations that have relatively few events need to have a very large sample size to be able to measure the magnitude of relative risks observed in the high-risk groups (relative risk of 1.5 to 2.5). In such a context, markers of subclinical atherosclerosis are important outcomes to examine. The detection of coronary artery calcification (CAC) by ultrafast computed tomography (CT) scanning is highly predictive of the presence of histopathologic atherosclerosis [3], and the extent of calcification correlates well with plaque burden [4]. It is also an accurate (positive predictive value of 84% to 96%) measure of obstructive CAD compared with angiographic evaluation and is a useful tool to study subclinical CAD, especially in population settings [5]. Some argue that, in the setting of observational studies, CAC measurement may even be superior to other measures such as carotid intima-media thickness in predicting cardiovascular outcomes [6].

The primary objective of this epidemiological study was to understand the relationship between sUA concentration and CAC in relatively young and healthy adults. If the hyperuricemia-CAD link is real, we can expect that the prevalence of CAC among those with higher sUA levels will be greater and that the extent of CAC will be directly proportional to the degree of hyperuricemia - a hypothesis that we tested here.

## Materials and methods

### Design

We performed cross-sectional analyses of year-15 data from the Coronary Artery Risk Development in Young Adults (CARDIA) study, a prospective observational cohort study of 5,115 subjects recruited between the ages of 18 and 30 years and followed for 15 years. Ethical approval for the CARDIA study was obtained from participating institutions, and informed consent was obtained from the patients.

### Setting, participants, and follow-up

The CARDIA study is an ongoing multicenter cohort study based at four centers: Chicago, IL; Birmingham, AL; Minneapolis, MN; and Oakland, CA. The observation baseline of this study was 1985-1986, when all participants were recruited and enrolled. The cohort had approximately equal numbers of African-Americans and whites, men and women, adults 18 to 24 years old and 25 to 30 years old, and participants with more than and less than high school education [7]. Subsequently, they were followed up at years 2, 5, 7, 10, 15, and 20. A detailed description of the study methodology has been published [7]. At baseline and every follow-up visit, CARDIA study participants underwent extensive medical examinations with a specific focus on cardiovascular risk factors. The study provided detailed information on demographic characteristics and on lifestyle habits such as alcohol consumption and smoking.

### Inclusion and exclusion criteria

We studied the data collected during year 15 of this prospective study, at which time all participants were invited to obtain an electron beam computerized tomography (EBCT) scan. We excluded all participants with missing values for CAC scores or sUA concentrations and any self-reported coronary heart disease, including angina symptoms. Since diabetes is associated with both higher sUA concentration and higher incidence rates of CAC [8], we excluded all subjects with type 2 diabetes or prediabetes (defined by American Diabetes Association criteria [9]) and those who reported the use of diabetes medications or a physician diagnosis of diabetes. Since the presence of renal impairment can affect sUA concentration and atherosclerosis, individuals with an estimated glomerular filtration rate of less than 60 mL/minute per 1.73 m^2 ^(calculated by the Modification of Diet in Renal Disease equation) were also excluded [10].

### Coronary artery calcification measurement, case definition, and rationale

In a single session, two CT scans were obtained at year 15 for each participant by using an EBCT scanner (Imatron C-150™; GE Medical Systems, Milwaukee, WI [Chicago and Oakland centers]) or a multidetector CT scanner (GE Lightspeed™; GE Medical Systems [Birmingham center] or Volume Zoom™; Siemens, Erlangen, Germany [Minneapolis center]). Details of the CT protocol have been published [11]. The amount of CAC can be measured to provide a reasonable estimate of total coronary atheroma, including calcified and non-calcified plaque. Coronary calcium assessments for diagnosis of atherosclerosis and obstructive disease and for risk stratification for future cardiac events have undergone significant validation over the past 20 years [12,13]. The extent of calcification was quantified by using the Agatston method, in which total calcium scores were calculated on the basis of the number, areas, and peak Hounsfield computed tomographic numbers of the calcific lesions [4]. A previous study showed that an Agatston score of zero indicates no identifiable plaque with a negative predictive value of 98% for those 40 to 49 years old, an age group similar to that of our cohort [4]. In angiographic studies done in older populations, scores of 1 to 99 indicate mild plaque, 100 to 399 moderate plaque, and at least 400 severe atherosclerotic plaque burden. Given that our goal was to assess for any CAC in young adults with no clinical evidence of CAD, we defined CAC as any positive, non-zero Agatston score, using the average of two scans. Each scan with at least one non-zero score (*n *= 350, 11.5%) was reviewed by an expert investigator who was blinded to the scan scores to verify CAC presence. The agreement between scans was high (kappa = 0.79, 95% confidence interval [CI] 0.75 to 0.83), and discordance was only 3.6% [14].

### Serum uric acid

Fasting concentration of sUA was measured in a central laboratory by using a colorimetric assay with rigorous quality control.

### Statistical analyses

Our primary outcome measure was subclinical atherosclerosis, defined as the presence of CAC by CT scan. Several cutoff points ranging from 0 to 1,000 have been used in other studies to define the presence of CAC, depending on its prevalence. We assessed the CT images for evidence of any CAC (defined as Agatston score of greater than 0) and for the presence of at least mild plaque (Agatston score of greater than 10). The choice of these cutoffs was dictated by the statistical distribution of Agatston score in our young population.

The first objective was to examine the relationship between measures of subclinical atherosclerosis and sUA (as a continuous as well as a stratified measure). These analyses were performed by using logistic regression models in which presence or absence of CAC (defined as an Agatston score of greater than 0 or greater than 10) was the dependent variable and sUA was the independent variable of interest. sUA quartiles were defined for men and women separately and were subsequently pooled.

We adjusted for the following covariates measured at year 15: age, gender, race, high- and low-density lipoproteins, triglycerides, smoking, blood pressure stage [15], presence of metabolic syndrome [16], C-reactive protein, waist circumference, alcohol use, creatinine, and serum albumin concentration. These factors have been assessed in previous studies of hyperuricemia and cardiovascular risk and therefore were included in the present analyses. In our primary analyses, all of these covariates were included in the model, regardless of the statistical significance of each. Subsequent confirmatory analyses deployed backward selection methods (with *P *< 0.20 as the cutoff) to derive a more parsimonious model.

The second objective of the analyses was to test the hypothesis that, among those with CAC, Agatston scores will be directly proportional to sUA concentration. The distribution of these scores was skewed, with numerous outliers. Hence, in these analyses, we used ordinary least square (OLS) regression models in which the dependent variable was the log-transformed Agatston score (log_2 _[CAC + 1]). For these OLS regression analyses, we excluded all participants who had an Agatston score of zero.

In all regression models, we explored the data for the presence of statistical interaction between gender, race, and sUA. Model fit was verified by using the Hosmer-Lemeshow method [17]. Data analyses were performed by using SAS^® ^(SAS Institute Inc., Cary, NC, USA).

## Results

Of the 5,115 participants at baseline, 3,671 participated in the examination at year 15. Among these, 1,173 were excluded as they met the study exclusion criteria. Overall, there were 2,498 participants (1,211 men and 1,287 women) available for analyses. Table 1 shows the characteristics of these participants. Higher sUA concentrations were associated with greater prevalence of cardiovascular risk factors, metabolic syndrome, and high Framingham risk score (Table 1). Fewer than 20 participants had a self-reported history of probable or definite gout and these could not be independently verified. None of the study population was using urate-lowering medications such as allopurinol, probenecid, sulfinpyrazone, or losartan.

**Table 1:** Characteristics of study population by gender and serum uric acid quartile

	Women (*n *= 1,287)				Men (*n *= 1,211)			
	Quartile 1	Quartile 2	Quartile 3	Quartile 4	Quartile 1	Quartile 2	Quartile 3	Quartile 4
sUA range, μmol/L [mg/dL]	77-196 [1.3-3.3]	196-226 [3.3-3.8]	232-274 [3.9-4.6]	280-636 [4.7-10.7]	155-291 [2.6-4.9]	297-333 [5.0-5.6]	339-393 [5.7-6.6]	399-690 [6.7-11.6]
Age, years	40.1 (3.8)	40.1 (3.6)	40.3 (3.6)	40.5 (3.8)	40.1 (3.6)	39.8 (3.5)	40.4 (3.5)	40.4 (3.6)
African-American	43.5%	42.6%	46.5%	57.5%	42.7%	37.4%	42.5%	43.2%
Body mass index, kg/m^2^	24.8 (4.7)	27.2 (6.2)	29.6 (6.8)	33.1 (7.2)	26.1 (3.8)	27.4 (4)	28.1 (4.5)	30.1 (4.6)
Alcohol, mL/day	6.2 (10)	5.7 (11.8)	9 (32.7)	9.1 (22)	13.3 (35.4)	14.2 (25.1)	16.2 (24.4)	20.3 (35.1)
Smokers	36.6%	39.7%	38%	46.2%	35.3%	39.4%	39.3%	40.2%
Systolic BP, mm Hg	107 (12.5)	109.3 (14)	112.4 (15.3)	115.5 (16.3)	111.7 (11.2)	114.8 (13.5)	115.4 (12.9)	118.3 (14.7)
Diastolic BP, mm Hg	69.6 (10.1)	71.5 (10.2)	73.3 (10.9)	75.9 (12.6)	73.3 (9.2)	76.3 (11)	76.8 (10.2)	79.2 (12.1)
Serum fasting glucose, mmol/L [mg/dL]	4.45 (0.40) [80.2 (7.2)]	4.48 (0.43) [80.7 (7.7)]	4.5 (0.45) [81.1 (8.1)]	4.7 (0.53) [84.3 (9.5)]	4.7 (0.46) [84 (8.3)]	4.8 (0.53) [85.7 (9.6)]	4.8 (0.52) [86.4 (9.4)]	4.9 (0.54) [88.8 (9.7)]
Serum HDL-C, mmol/L [mg/dL]	1.54 (0.33) [59.7 (12.8)]	1.44 (0.35) [55.5 (13.5)]	1.45 (0.36) [56 (14.1)]	1.32 (0.37) [51 (14.4)]	1.24 (0.33) [48 (12.9)]	1.19 (0.32) [45.9 (12.4)]	1.14 (0.31) [43.9 (11.8)]	1.10 (0.33) [42.4 (12.6)]
Serum LDL-C, mmol/L [mg/dL]	2.72 (0.73) [105.1 (28.2)]	2.74 (0.74) [105.9 (28.7)]	2.80 (7.7) [108.4 (29.8)]	3.01 (0.79) [116.3 (30.6)]	2.87 (0.74) [110.9 (28.5)]	3.12 (0.81) [120.5 (31.5)]	3.19 (0.95) [123.5 (36.6)]	3.23 (0.92) [125 (35.7)]
Serum triglycerides, mmol/L [mg/dL]	0.78 (0.38) [68.8 (33.8)]	0.89 (0.44) [78.7 (39.1)]	0.95 (0.52) [84.5 (45.8)]	1.27 (0.80) [112.6 (70.7)]	1.01 (0.67) [89.8 (59.7)]	1.26 (0.76) [111.5 (67.4)]	1.52 (1.87) [135 (165.7)]	1.86 (1.76) [164.8 (156.1)]
Serum creatinine, μmol/L [mg/dL]	79.6 (8.8) [0.9 (0.1)]	79.6 (8.8) [0.9 (0.1)]	79.6 (8.8) [0.9 (0.1)]	79.6 (8.8) [0.9 (0.1)]	97.2 (8.8) [1.1 (0.1)]	97.2 (8.8) [1.1 (0.1)]	97.2 (17.7) [1.1 (0.2)]	97.2 (17.7) [1.1 (0.2)]
Waist circumference, cm	76.6 (10.1)	81.6 (12.1)	86.3 (13.1)	94 (14.1)	87.8 (9.5)	91.8 (9.6)	93.5 (11)	98.4 (10.9)
eGFR, abbreviated MDRD	87.2 (15.1)	85.6 (15.5)	83.9 (14.2)	82.7 (15.2)	89.9 (13.6)	88.3 (14.1)	87.1 (19.3)	85.6 (16.1)
Framingham risk score	-4.8 (4.1)	-3.7 (4.3)	-3.3 (4.4)	-1.4 (4.3)	1.1 (2.2)	1.8 (2.4)	2.3 (2.3)	2.7 (2.4)
C-reactive protein, mg/dL	1.5 (2.9)	1.5 (1.7)	1.6 (2.9)	1.8 (1.7)	1.7 (2.9)	1.8 (2.0)	2.0 (2.5)	2.1 (2.5)

Data are presented as range, mean (standard deviation), or percentage. BP, blood pressure; eGFR, estimated glomerular filtration rate; HDL-C, high-density lipoprotein cholesterol; LDL-C, low-density lipoprotein cholesterol; MDRD, modification of diet in renal disease; sUA, serum uric acid.

The mean ± standard deviation of sUA concentration was 345 ± 77 μmol/L (5.8 ± 1.3 mg/dL) for men and 238 ± 65 μmol/L (4.0 ± 1.1 mg/dL) for women (*P *< 0.001). sUA was distributed normally among men and women, whites, and African-Americans. The proportions of participants with sUA of greater than 416 μmol/L (7.0 mg/dL) were 8.4% (*n *= 211) overall, 16.6% (*n *= 201) among men, and 0.8% (*n *= 10) among women. The men had a significantly worse overall cardiovascular risk profile than women (Table 1). sUA concentrations were correlated with male gender (correlation coefficient of 0.6; *P *< 0.01) and Framingham risk score (0.52; *P *< 0.01) but were only modestly associated with other known cardiovascular risk factors (all correlation coefficients of less than 0.3; *P *< 0.05). C-reactive protein levels were not correlated with sUA (*P *= 0.20).

The majority (90.5%, *n *= 2,260) of participants had no detectable CAC. Overall, 9.5% (*n *= 238) of participants had an Agatston score of greater than 0, 6.3% (*n *= 158) had an Agatston score of greater than 10, and 1.4% (*n *= 34) had an Agatston score of greater than 100. As expected in a cohort free of clinical CAD, relatively few participants had an Agatston score of greater than 400 (*n *= 4). Among those with any CAC, mean scores were higher in men than in women (75.0 versus 60.8; Wilcoxon rank sum test *P *= 0.77) and in whites than in African-Americans (71.6 versus 70.0; *P *= 0.19), but these findings were not statistically significant.

**Figure 1:**
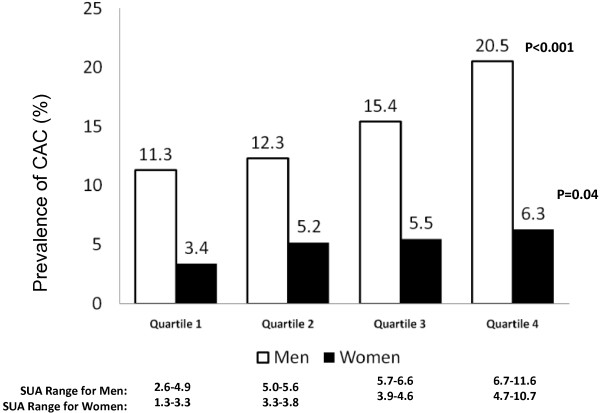
**Prevalence of any coronary artery calcification (Agatston score >0) by serum uric acid concentration among participants in the CARDIA study cohort at year 15**. A detailed description of these patients (1,211 men and 1,287 women) is provided in Table 1. *P *values are for trend test. CAC, coronary artery calcification; CARDIA, Coronary Artery Risk Development in Young Adults; SUA, serum uric acid.

At each quartile of sUA, men had a greater prevalence of CAC than women (Figure 1). However, the prevalence of CAC increased with increasing sUA in both genders, and the highest quartile had almost two times the prevalence compared with the lowest (odds ratio [OR] 1.87, CI 1.19 to 2.93) (Table 2).

**Table 2:** Crude risk of increasing serum concentrations of uric acid

		Odds ratio for outcome			
	Serum uric acid concentration, μmol/L [mg/dL]	Agatston score >0 vs. Agatston score = 0		Agatston score >10 vs. Agatston score <10	
Men (*n *= 1,211)					
Quartile 1	155-291 [2.6-4.9]	1		1	
Quartile 2	297-333 [5.0-5.6]	1.17	(0.71-1.95)	1.21	(0.65-2.23)
Quartile 3	339-393 [5.7-6.6]	1.56	(0.96-2.54)	1.62	(0.91-2.91)
Quartile 4	399-690 [6.7-11.6]	2.07	(1.30-3.31)	2.08	(1.19-3.67)
Women (*n *= 1,287)					
Quartile 1	77-196 [1.3-3.3]	1		1	
Quartile 2	196-226 [3.3-3.8]	1.50	(0.66-3.38)	1.49	(0.52-4.22)
Quartile 3	232-274 [3.9-4.6]	1.44	(0.65-3.23)	1.5	(0.54-4.17)
Quartile 4	280-636 [4.7-10.7]	2.47	(1.17-5.22)	2.93	(1.15-7.49)
Overall (*n *= 2,498)					
Quartile 1	77-291 [1.3-4.9]	1		1	
Quartile 2	196-333 [3.3-5.6]	1.25	(0.81-1.91)	1.27	(0.75-2.15)
Quartile 3	232-393 [3.9-6.6]	1.47	(0.98-2.22)	1.54	(0.93-2.54)
Quartile 4	280-690 [4.7-11.6]	2.11	(1.42-3.12)	2.24	(1.39-3.60)

In the bivariate logistic regressions in which presence or absence of CAC was the dependent variable, the highest quartile of sUA concentrations (greater than 393 μmol/L [6.6 mg/dL] for men and greater than 274 μmol/L [4.6 mg/dL] for women) had an OR of greater than 2.0 among both men and women, regardless of the Agatston score cutoff used to define CAC (Table 3).

**Table 3:** Adjusted relative risk for subclinical atherosclerosis according to strata of serum uric acid concentrations

		Odds ratio for outcome	
	Serum uric acid concentration, μmol/L [mg/dL]	Agatston score >0 vs. Agatston score = 0	Agatston score >10 vs. Agatston score <10
Quartile of serum uric acid^a^			
Quartile 1	77-291 [1.3-4.9]	1	1
Quartile 2	196-333 [3.3-5.6]	1.24 (0.78-1.97)	1.26 (0.72-2.22)
Quartile 3	232-393 [3.9-6.6]	1.42 (0.9-2.24)	1.50 (0.87-2.58)
Quartile 4	280-690 [4.7-11.6]	1.87 (1.19-2.93)	1.91 (1.12-3.26)

Adjusted for the effects of age, gender, race, high- and low-density lipoproteins, triglycerides, smoking, blood pressure class, presence of metabolic syndrome, C-reactive protein, waist circumference, alcohol use, creatinine, and serum albumin concentration. No participants had diabetes or renal impairment. ^a^Men and women were classified into quartiles by gender-specific cutoff numbers and were subsequently pooled.

We developed parallel multivariable logistic regression models that included all of the risk factors of interest (age, gender, race, high- and low-density lipoproteins, triglycerides, smoking, blood pressure class, presence of metabolic syndrome, C-reactive protein, waist circumference, alcohol use, creatinine, and serum albumin concentration) in the model. Data were pooled for men and women, and gender-specific stratification for quartiles of sUA was used after we established that there was no statistically significant interaction between gender and the correlation between sUA concentration and CAC (Table 2). Although these models differed in the definition of subclinical atherosclerosis and in the stratification strategy for sUA, all models showed that the highest quartile of sUA concentration was associated with significantly higher risk for subclinical atherosclerosis. In both multivariable regression models, each unit increase in sUA concentration was associated with an OR of 1.23 (CI 1.09 to 1.39) for subclinical atherosclerosis. The findings were replicated in backward stepwise selection models that eliminated those factors that were not significant individually as well as those in which an alternate stratification strategy for sUA was used (data not shown).

**Figure 2:**
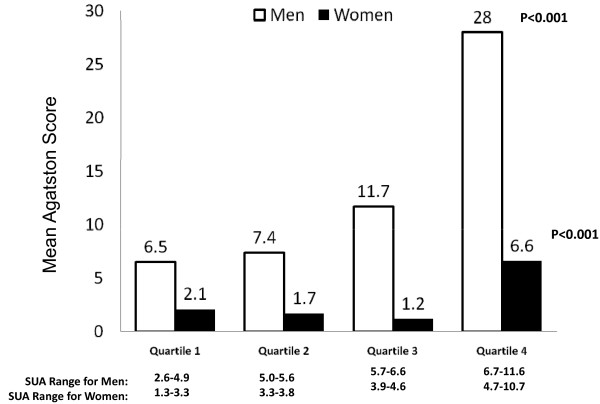
**Relationship between burden of coronary artery calcification (unmodified Agatston score) and serum uric acid concentrations**. These analyses included only those subjects who had an Agatston score of greater than zero (*n *= 238). *P *values are for trend test. SUA, serum uric acid.

The last set of analyses focused on the association between sUA concentration and the severity of CAC. These analyses included only those subjects who had Agatston score of greater than zero (*n *= 238). Although the Agatston scores were higher among those with higher sUA concentrations (Figure 2), bivariate correlation analyses showed that the association was not strong (correlation coefficient of 0.13). However, in multivariable OLS regression models, each unit increase in sUA concentration was associated with a significant increase in the log-transformed Agatston score (beta coefficient of 0.288, 95% CI 0.078 to 0.498; *P *= 0.008; R^2 ^= 0.197%). In other words, there was an approximately 22% increase in Agatston score for each unit increase in sUA. When examined separately for each gender, this association persisted for men (beta coefficient of 0.300, CI 0.078 to 0.522) and women (beta coefficient of 0.318, CI -0.502 to 1.138) but was statistically significant only in the former (*P *= 0.009).

## Discussion

The association between hyperuricemia and the presence of subclinical atherosclerosis has not previously been studied in a cohort of young adults with no risk factors for CAD. Our study found a direct correlation between the prevalence and severity of CAC and sUA concentration in both men and women. This supports the hypothesis that uric acid may be involved in the pathologic process of atherosclerosis independently of conventional risk factors.

Uric acid is a ubiquitous antioxidant in the blood [18]. Abnormally high serum concentrations of uric acid indicate oxidative stress, endothelial dysfunction, and slow coronary artery blood flow [19,20]. Elevated sUA concentration signifies a milieu with high oxidative stress and potentially indicates a vascular pathologic process such as atherosclerosis [21]. An association between hyperuricemia and CAC has been observed in previous studies involving patients with underlying risk factors for CAD such as type 1 diabetes, longstanding hypertension, or metabolic syndrome [22-25]. Importantly, a cross-sectional analysis of 443 individuals with type 1 diabetes suggested that the chances of progressive CAC were proportional to the magnitude of sUA concentration [22]. Another study involved older age groups compared with our cohort but also found that the correlation between uric acid and CAC was evident among men and women and in similar magnitude [13]. In the INSIGHT (International Nifedipine Study Intervention as Goal for Hypertension Therapy) study, in which CAC was measured in hypertensive patients who were older than 55 years of age and who had at least one more major cardiovascular risk factor, those with a total coronary calcium score (TCS) of greater than zero had a slightly higher sUA concentration compared with those with a TCS of zero (333 ± 89 versus 315 ± 83 μmol/L [5.6 ± 1.5 versus 5.3 ± 1.4 mg/dL]; *P *= 0.03) [23]. However, other studies, including the GENOA (Genetic Epidemiology Network of Arteriopathy) study on sibships with at least two members with diagnosed hypertension, did not show an association between uric acid concentration and the presence or severity of CAC after adjustment for conventional risk factors [25-27]. In addition, the National Heart, Lung and Blood Institute (NHLBI) Family Heart Study did not find a significant relationship between hyperuricemia and CAC in either gender [28]. Among those who underwent coronary angiography for suspected CAD, sUA concentration of greater than 416 μmol/L (7.0 mg/dL) was associated with stable plaques without evidence of remodeling. The authors interpret this as suggesting that uric acid is a marker of atherosclerosis rather than a pathogenic mediator [13,29].

Coronary atherosclerosis is less likely to be associated with calcification among women compared with coronary atherosclerosis with a similar degree of lumen narrowing in men [30]. In the CARDIA study cohort, the prevalences of CAC were approximately 15% among men and approximately 5% among women overall [14]. This could be because younger women may be 'resistant' to atheroma growth [31].

Gender might be an important effect modifier in the association between hyperuricemia and CAC because of differences in (a) the distribution of sUA and (b) the prevalence of CAC. Iribarren and colleagues [32] analyzed data from the Atherosclerosis Risk in Communities (ARIC) study and concluded that an association between sUA and cardiovascular risk is evident in men but not women. In contrast, a similar study by Ishizaka and colleagues [33] reported that gender was not a factor. Since the prevalences of hyperuricemia and CAC are both lower among women, a greater sample size would be needed to detect a given effect size of hyperuricemia-CAC association. We defined quartiles separately for men and women prior to pooling. Statistical tests of gender-sUA interaction were not significant in our data. In gender-specific analyses, the direction and magnitude of risk among women were similar to those among men; however, the standard errors were wide because of the lower power for precise estimates.

The major strength of our community-based study is the generalizability of our results to young adults, including men, women, African-Americans, and whites. All of the studies described earlier were performed among patients with greater-than-normal cardiovascular risk, such as those with hypertension, diabetes, metabolic syndrome, psoriasis, or renal disease [13,21,23,26-29].

The primary limitation of this study was the cross-sectional nature of data analysis. Survivor bias can affect cross-sectional data analyses in that those with more severe disease die prior to the time point of analysis; however, this is not a major consideration in the CARDIA study as the main cause of mortality in the first 16 years was non-cardiovascular in the vast majority of patients (117/127 deaths out of 5,115 enrollees). Our future studies will examine the rate of progression of CAC over time among patients with hyperuricemia or gout or both. Gouty arthritis has been associated with CAD among middle-aged men [34], but our study had too few participants with gout (n <20) to allow a formal analysis. A larger number of women would have enabled separate analyses with respect to use of exogenous hormones and menstrual status. Owing to the design of our study, there was relatively little heterogeneity with respect to age (approximately 12 years), precluding an analysis of impact of age on the hyperuricemia-CAC association. However, in our analyses, age was not significantly associated with sUA concentration. sUA concentration is known to vary with time of day and recent dietary intake and possibly with physical exertion, introducing 'random noise' in sUA data. Unless sUA increases due to such variables can be shown to occur preferentially among those with higher CAC scores, this issue cannot explain our findings. As in all other epidemiological studies, unmeasured covariates could have caused residual confounding in our study as well.

## Conclusions

We have shown for the first time that sUA is associated with the presence and severity of CAC in young healthy adults, implicating a potential role of uric acid in the pathogenesis of subclinical atherosclerosis. Our data are consistent with the growing body of literature that implicates the vascular injury associated with hyperuricemia - both macrovascular and microvascular [2,22,35].

## Abbreviations

CAC: coronary artery calcification; CAD: coronary artery disease; CARDIA: Coronary Artery Risk Development in Young Adults; CI: confidence interval; CT: computed tomography; EBCT: electron beam computerized tomography; OLS: ordinary least square; OR: odds ratio; sUA: serum uric acid; TCS: total coronary calcium score.

## Competing interests

EK has consultant/advisor/grant recipient relationships with Takeda Pharmaceuticals International, Inc. (Deerfield, IL, USA). He has been a shareholder of Savient Pharmaceuticals, Inc. (East Brunswick, NJ, USA) and currently holds common stock in that company. He is an investigator for a clinical trial performed by Ardea Biosciences (San Diego, CA, USA). He serves on advisory boards for Takeda Pharmaceuticals International, Inc., URL Pharma (Philadelphia, PA, USA), and UCB (Brussels, Belgium). BJP and OD are employees of Takeda Pharmaceuticals International, Inc. LC declares that she has no competing interests.

## Authors' contributions

EK conceived of the manuscript idea, designed the analysis plan, performed statistical analysis, interpreted the results, and wrote the first draft of the manuscript with assistance from all other authors. He has possession of raw data sets and takes responsibility for the integrity of the data and the accuracy of the data analysis. Takeda Pharmaceuticals International, Inc. did not have access to the raw data, and Takeda Pharmaceuticals International, Inc. authors (BJP and OD) contributed primarily to refinement of study design, interpretation of data, and editing and revising of the initial drafts. All authors read and approved the final manuscript.
